# High resolution XUV Fourier transform holography on a table top

**DOI:** 10.1038/s41598-018-27030-y

**Published:** 2018-06-06

**Authors:** G. K. Tadesse, W. Eschen, R. Klas, V. Hilbert, D. Schelle, A. Nathanael, M. Zilk, M. Steinert, F. Schrempel, T. Pertsch, A. Tünnermann, J. Limpert, J. Rothhardt

**Affiliations:** 1 Helmholtz-Institute Jena, Fröbelstieg 3, 07743 Jena, Germany; 2 Institute of Applied Physics, Abbe Center of Photonics, Friedrich Schiller University Jena, Albert-Einstein-Straße 15, 07745 Jena, Germany; 3 Fraunhofer Institute for Applied Optics and Precision Engineering, Albert-Einstein-Str. 7, 07745 Jena, Germany

## Abstract

Today, coherent imaging techniques provide the highest resolution in the extreme ultraviolet (XUV) and X-ray regions. Fourier transform holography (FTH) is particularly unique, providing robust and straightforward image reconstruction at the same time. Here, we combine two important advances: First, our experiment is based on a table-top light source which is compact, scalable and highly accessible. Second, we demonstrate the highest resolution ever achieved with FTH at any light source (34 nm) by utilizing a high photon flux source and cutting-edge nanofabrication technology. The performance, versatility and reliability of our approach allows imaging of complex wavelength-scale structures, including wave guiding effects within these structures, and resolving embedded nanoscale features, which are invisible for electron microscopes. Our work represents an important step towards real-world applications and a broad use of XUV imaging in many areas of science and technology. Even nanoscale studies of ultra-fast dynamics are within reach.

## Introduction

High-resolution imaging is an indispensable tool in fundamental science as well as in technical and commercial applications for understanding the properties of samples under consideration. Using photons, rather than electrons, for imaging in the nanoscale offers the advantage of higher penetration depth in addition to chemical sensitivity and spectroscopic features^1,2^. Because the achievable resolution scales with the wavelength, light sources in the XUV and X-ray spectral range are necessary for the highest resolution. Unfortunately, in conventional X-ray microscopes, the resolution is not limited by the wavelength of the source alone but rather by the quality of optics used for focusing and/or image formation^3–5^. To overcome limitations imposed by X-ray optics, the intensity distribution of the light scattered from the sample can directly be measured on a pixelated detector and the missing phase information can be retrieved by using phase retrieval algorithms^6^. Using these techniques, collectively known as coherent diffractive imaging (CDI), two-dimensional and three-dimensional resolutions of ~5 nm^7,8^ have been demonstrated employing synchrotrons and free-electron lasers as light sources. Such imaging techniques are ideally suited to e.g. uncover the nanoscale details of biological cells without time-consuming sectioning^9^ or to image the complete 3D structure of modern semiconductor integrated circuits^10^. Moreover, they can map the chemical composition e.g. of a Li battery^7^, nanomaterials or functional polymers on smallest scales. In combination with ultra-short pulses and pump-probe experiments, ultrafast dynamics of magnetization, heat- or energy- transport can also be observed^11,12^.

So far, the broad use of these powerful imaging techniques in science and technology has mainly been hindered by the fact that suitable table-top light sources have not been available and thus coherent X-ray imaging has been limited to large scale facilities. Clearly, a laboratory-scale alternative is required to exploit the full potential of coherent XUV- and X-ray imaging. In recent years, laser driven high-order harmonic generation (HHG) sources have demonstrated the generation of coherent XUV light with a photon flux up to 10^14^ photons/second^13–15^. Employing these table-top sources for phase-retrieval-based CDI enabled impressive sub-wavelength resolutions of ~13 nm^16,17^ in a transmission geometry. Phase retrieval algorithms employed in conventional CDI start by applying random phase to the measured intensity pattern and utilize additional constraints to converge iteratively to the correct phase distribution. This takes a lot of computational effort and convergence is strongly dependent upon coherence of the source, linearity of the detector, oversampling ratio and signal-to-noise ratio (SNR) of the measured data^18^.

In Fourier transform holography (FTH), the phase of the diffracted light from the object is encoded directly on the far-field interference pattern by adding a suitable reference wave generated by a hole or a localized scatterer located in close proximity of the imaged object^19^. The amplitude and phase profile of the object is then recovered from the cross-correlation terms by computing the Fourier transform of the measured hologram. The resolution in this case depends on the size of the reference structures in addition to the coherence and photon energy of the source, and the numerical aperture (NA) of the measurement^19,20^. Reduction of the reference structure size improves resolution but at the cost of image contrast. Usage of multiple reference structures [32] and uniformly redundant arrays [27] are among the methods proposed to improve image SNR of conventional FTH. A subsequent use of phase retrieval algorithms can lift the resolution limit imposed by the reference structure size altogether, provided that the measured diffraction pattern fulfils the oversampling criterion. A related holographic imaging technique that employs extended references, like slits or squares, was shown to increase image contrast and potentially enhance resolution^21–23^. However, the additional differentiation operation required by these techniques tends to amplify the noise at higher spatial frequencies especially in the photon-flux-limited regime^24,25^. Furthermore, the resolution improvement in these techniques is achieved only in the specific direction of a sharp edge^22^. Previous works on FTH at synchrotrons achieved half-pitch resolutions of ~50 nm, which were limited by the size of the reference structures^26,27^. Due to its robustness, this imaging method has nevertheless been used for groundbreaking studies of ultrafast magnetism on the sub-ps time scale^11,28^. Using table-top XUV/soft X-ray sources, the highest resolution obtained so far is 89 nm and the respective experiment required hours of acquisition time^29^.

In this work, a half-pitch resolution of 34 nm is achieved with just tens of seconds of acquisition time in a transmission type FTH geometry using reference structures having diameters of only 50 nm (2.7λ). The low transmission through the reference structures caused by waveguiding effects^30^ is compensated by the high photon flux source used in the experiment. Moreover, the smallest features ever imaged in a table-top XUV/soft X-ray setup are resolved by refining the result from FTH using iterative phase-retrieval algorithms. In addition, imaging of embedded features through a Si_3_N_4_ membrane is demonstrated, which would not be possible with an electron microscope. In imaging wavelength-scale features, we additionally show that waveguiding effects play an important role for image formation. Our approach provides an imaging platform offering highest resolution and robust image formation in amplitude and phase and thus qualifies for real-world samples such as weakly-scattering biological cells or complex multi-material integrated circuits. Our technique is readily operational in a table-top format and can potentially be transferred to many scientific laboratories, integrated into machines and even clinical applications appear feasible. Furthermore, the ultrashort pulse duration of the employed XUV source provides additional possibilities of observing ultrafast dynamics on the nanoscale.

## Results

**Figure 1:**
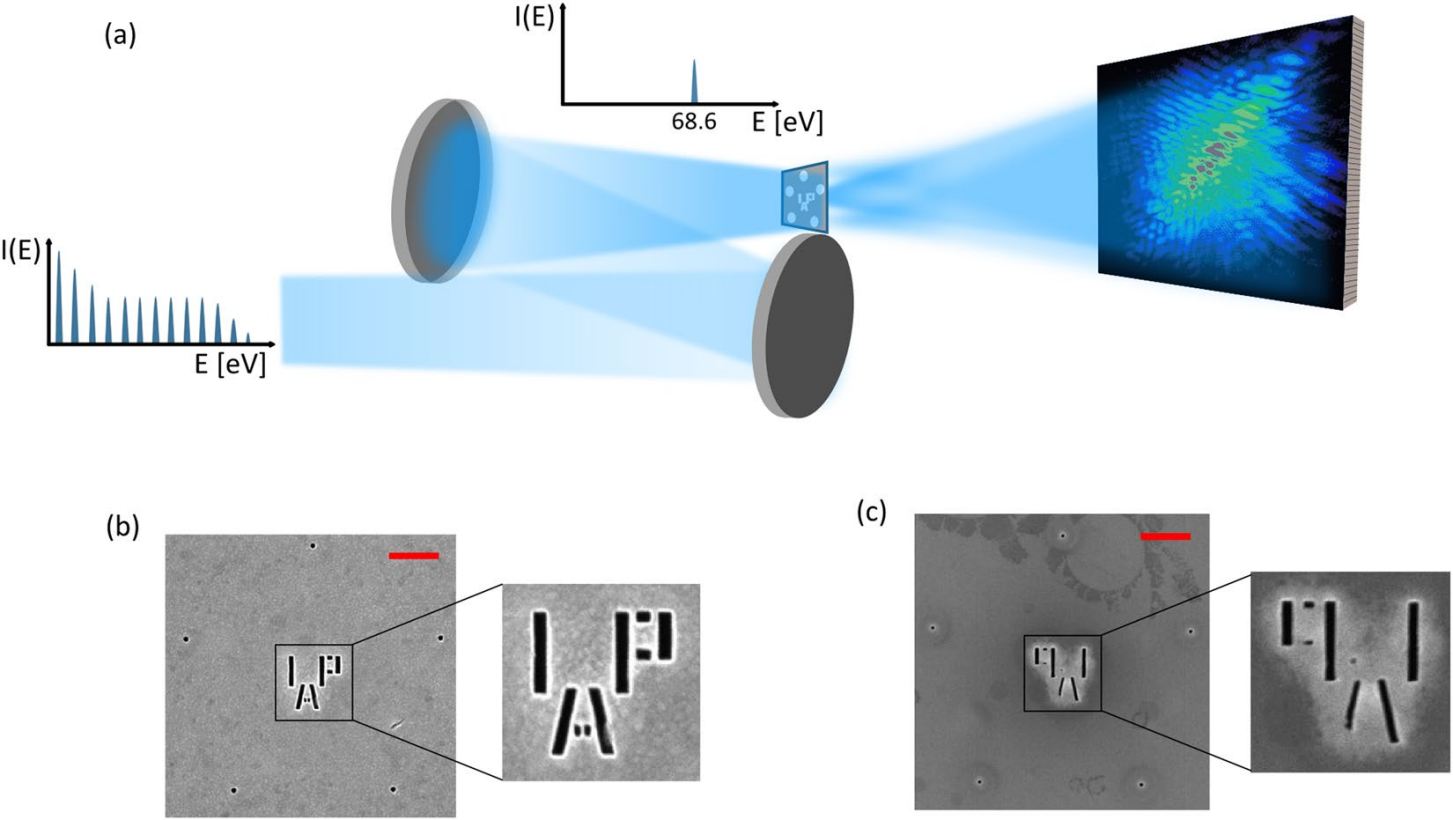
Schematic diagram of the experimental setup. (**a**) The high-order harmonic generation (HHG) source delivers a broad spectrum of harmonic lines. Two multilayer mirrors select a single harmonic at 68.6 eV while simultaneously focusing it at the sample. HIM image of (**b**) the sample’s front side facing a 200 nm thick gold layer and (**c**) the back side facing a 50 nm thick silicon nitride membrane. Scale bars in (**b**) and (**c**) are 500 nm. The energy of the Helium ions during imaging was 30 keV.

An HHG source generating harmonics from 55 eV–70 eV is used and a single harmonic line at 68.6 eV is selected by a pair of multilayer mirrors (see Methods). As shown in Fig. 1(a), the XUV beam is simultaneously focused by the multilayer mirrors onto the sample, which consists of a gold-coated Si_3_N_4_ membrane. The hologram created by the interference between waves from the sample and from the reference holes is recorded by an XUV-CCD camera in the far-field. Helium ion microscope (HIM) pictures of the front and back side of the sample show the region of interest in the center and five reference holes about 1.25 µm away from the center (Fig. 1(b,c)). The achievable resolution in FTH is ~70% of the reference hole diameter^31^ and, therefore, small reference holes are required for high resolution. Reducing the hole size, on the other hand, will result in a lower image contrast because of a reduced intensity of the reference wave at the detector^26^. In our work, a reference hole size of 50 nm was chosen as a compromise between the resolution and contrast of the imaging system. Precise fabrication of these small holes is challenging and tackled with milling using a HIM (see methods). Multiple reference holes allow increasing the SNR of the retrieved image and the diversity also helps to offset fabrication issues of individual holes while offering some statistics for image interpretation^32^. In Fig. 1(c) it can be seen that all five holes are completely drilled through the membrane, while at some features of the sample Au/Si_3_N_4_ layers were not completely etched through.

**Figure 2:**
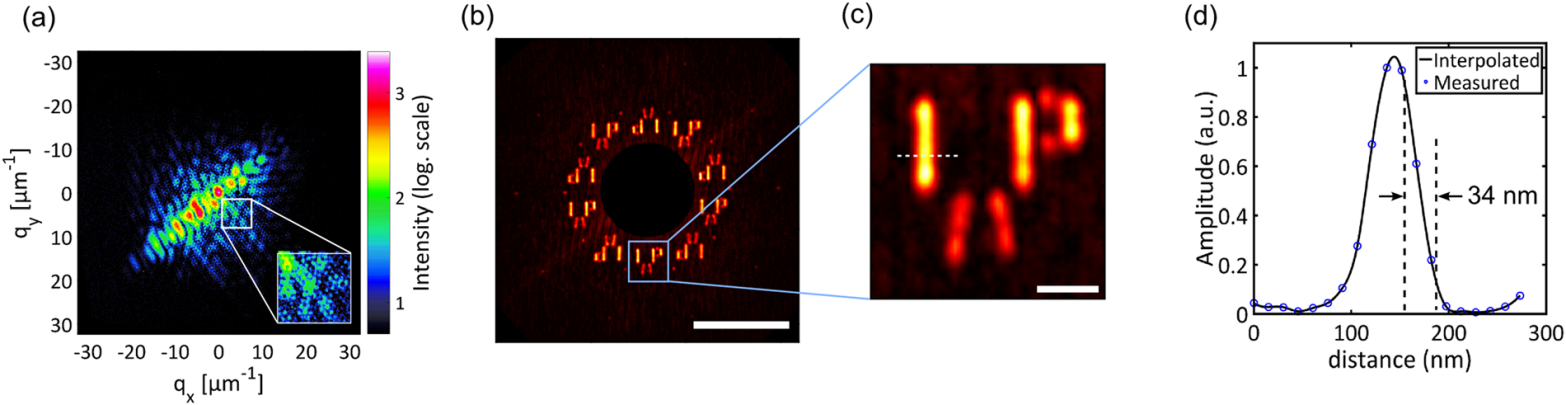
Results of the FTH experiment. (**a**) Recorded hologram showing good SNR until a momentum transfer (q_x_ and q_y_) of 20 µm^−1^ (**b**) Fourier transform of the hologram in (**a**) with the central autocorrelation blocked (scale bar is 2 µm). (**c**) The lowest replica of the sample among the five independent cross-correlations (scale bar is 200 nm). (**d**) Cross-section along the white line (in (**c**)) showing a half-pitch resolution of 34 nm.

Due to the high photon flux of our source, an acquisition time of 20 seconds was enough to record holograms with sufficiently high NA to reach the resolution limit set by the reference hole size. It can be seen in the hologram (Fig. 2(a)) that the diffraction pattern of the sample is modulated by the interference with the reference waves yielding a honey-comb type pattern. The diffraction spans spatial frequencies up to 20 µm^−1^ corresponding to a half-pitch resolution of 25 nm which is well below the theoretical limit of 70% of the reference hole size (35 nm). The Fourier transform of the measured hologram gives the autocorrelation including cross-correlation terms of the sample with the reference holes and is shown in Fig. 2(b). Note that the autocorrelation term in the center is suppressed, since it is much more intense than the relevant cross-correlation terms. Also, note that five cross-correlation terms and their complex conjugates are visible for the sample. The standard deviation among the five copies averaged over each pixel of the sample is found to be <15% for the amplitude and <π/20 for the phase demonstrating the robustness and reliability of image formation. The cause for the slight variation among the five replicas is the difference in the actual reference hole diameter as will be revealed later. The holographic image formed by the smallest reference hole provides the highest resolution^33^ and is thus displayed in Fig. 2(c). It contains all the features seen in the back side HIM picture of the sample shown in Fig. 1(c). A cross-section along the dotted horizontal line of Fig. 2(c) shows a half-pitch (10/90) resolution of 34 nm which is at the theoretical limit imposed by the reference hole size. This is the highest resolution obtained with FTH from any light source including synchrotrons and X-ray free electron lasers. Note that some of the previous works^29^ used intensity (rather thanamplitude) in determining resolution values which gives up to a factor of 1.4 better resolution estimate for the same data.

**Figure 3:**
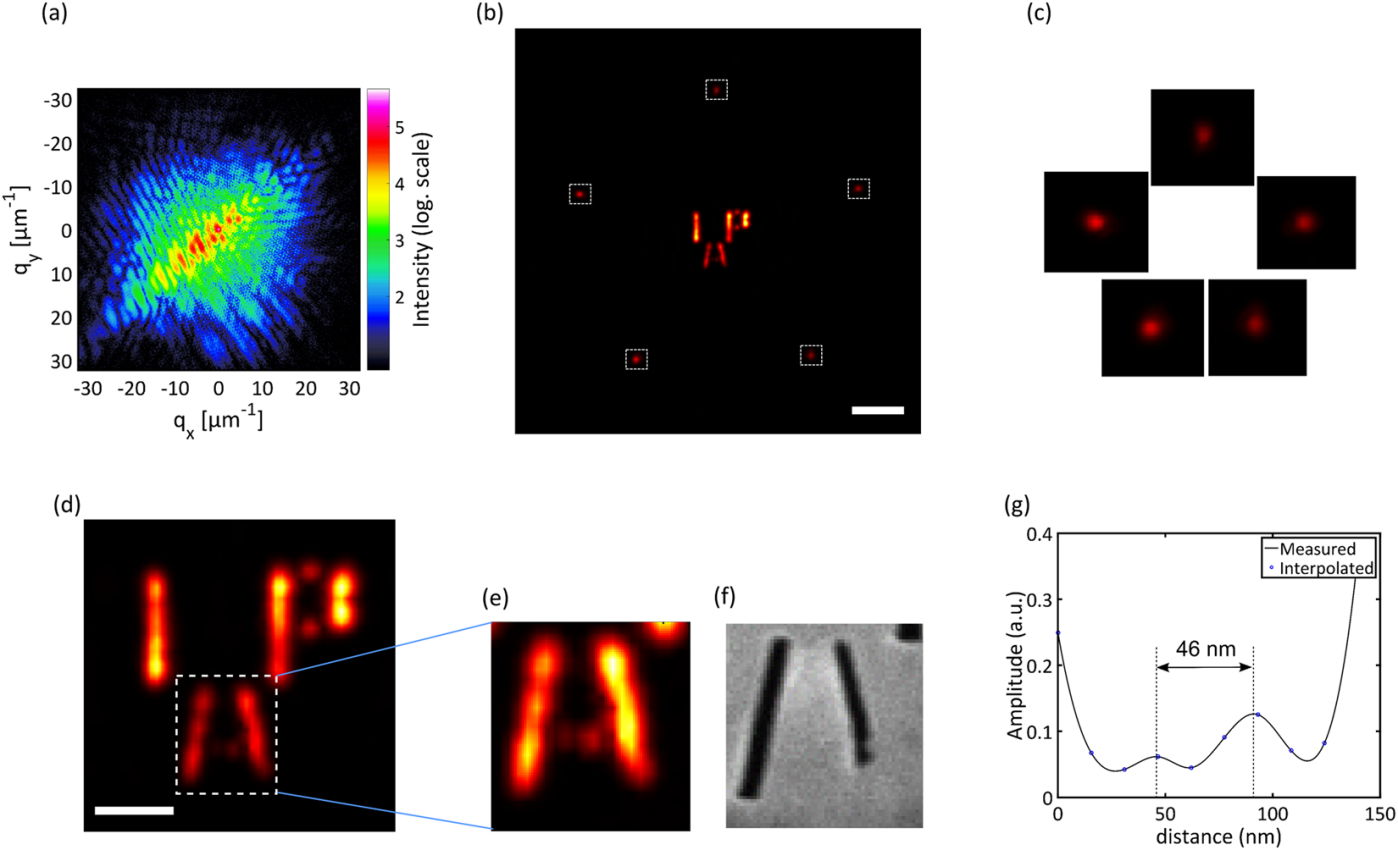
Refinement of resolution using phase retrieval algorithms (**a**) Recorded hologram with higher dynamic range (**b**) image of the sample and reference holes reconstructed with iterative phase retrieval using the image from FTH (Fig. 2c) as a seed (scale bar is 500 nm). (**c**) Zoomed-in image of the reference holes shown in their relative positions. (**d**) Zoomed-in image of the sample (scale bar is 200 nm). (**e**) An amplitude-rescaled image of the letter ‘A’. (**f**) The back side HIM picture showing the same region as (**e**). (**g**) Cross-section along the two dots of the letter “A” from(**e**).

The image quality and resolution can be further improved by performing iterative phase retrieval on the recorded hologram. In this case, a higher dynamic range dataset is constructed by merging two diffraction patterns with acquisition times of 60 seconds and 5 minutes displayed in Fig. 3(a). The measured diffraction has signal above the noise floor until the edge of the detector corresponding to an Abbe-limit of 15 nm. The guided variant of the relaxed averaged alternating reflections (RAAR) algorithm^34,35^ is used to perform the iterative phase retrieval (see Methods). The algorithm converges but a parametric run of the different parameters in the reconstruction and few thousands of iterations are necessary (this takes few minutes on a standard PC). In contrast, seeding the available image from holography already gives the algorithm the rough sample features, which normally take most of the time in a reconstruction. This increases the reliability of the reconstructions and a high-resolution image can be retrieved in a few hundred iterations (few seconds on a standard PC). Of course, the result shown in Fig. 3(b) has a better contrast and sharper edges than the result from FTH only. The amplitude variation along the longer bars of letters ‘I’ and ‘P’ in Fig. 3(d) nicely follows the width variation of these bars in the HIM picture of Fig. 1(c) with brighter parts corresponding to larger widths in the HIM picture. The close-up look of the reconstructed image of letter ‘A’ in Fig. 3(e) and its comparison with the same region from the back side HIM picture in Fig. 3(f) shows that the reconstruction reveals more than what is visible from the back side of the HIM picture. In particular, the two dots of the letter ‘A’ are not fully drilled through the membrane but are now visible in the reconstructed image. One of these dots is brighter although the HIM image of the front side shows dots of the same size. The higher transmission of the dot on the left side can thus be ascribed to a deeper drilling into the material. This demonstrates the penetrative capability of imaging with XUV sources in contrast to electron microscopy. In addition, a cross-section along these two dots of letter ‘A’ depicted in Fig. 3(g) shows that the half-distance between the two dots (and also between the dots and the neighboring legs of the letter ‘A’) is ~23 nm. As the dots are clearly separated, the resolving power of the imaging setup must be better than this value. These are the smallest features ever resolved in a table-top XUV/soft X-ray setup, in accordance with the classical Rayleigh criterion, in addition to being imaged through a membrane. Supplementary 3D information can be gained by estimating the thickness of the remaining gold/Si_3_N_4_ membrane from the measured transmitted intensity. This can be done by taking into account both absorption and damping of the waveguide modes although the qualitative information that the dot on the left side of Fig. 3(e) has more unetched gold/Si_3_N_4_ layer than the one on the right is apparent. Considering the transmittances of Si_3_N_4_ and gold at 18.1 nm, it is estimated that both dots in Fig. 3(e) have the 50 nm Si_3_N_4_ layer unetched. In addition, the dot on the left in Fig. 3(e) has an estimated 25 nm of gold layer while the one on the right has 8 nm of gold layer left.

**Figure 4:**
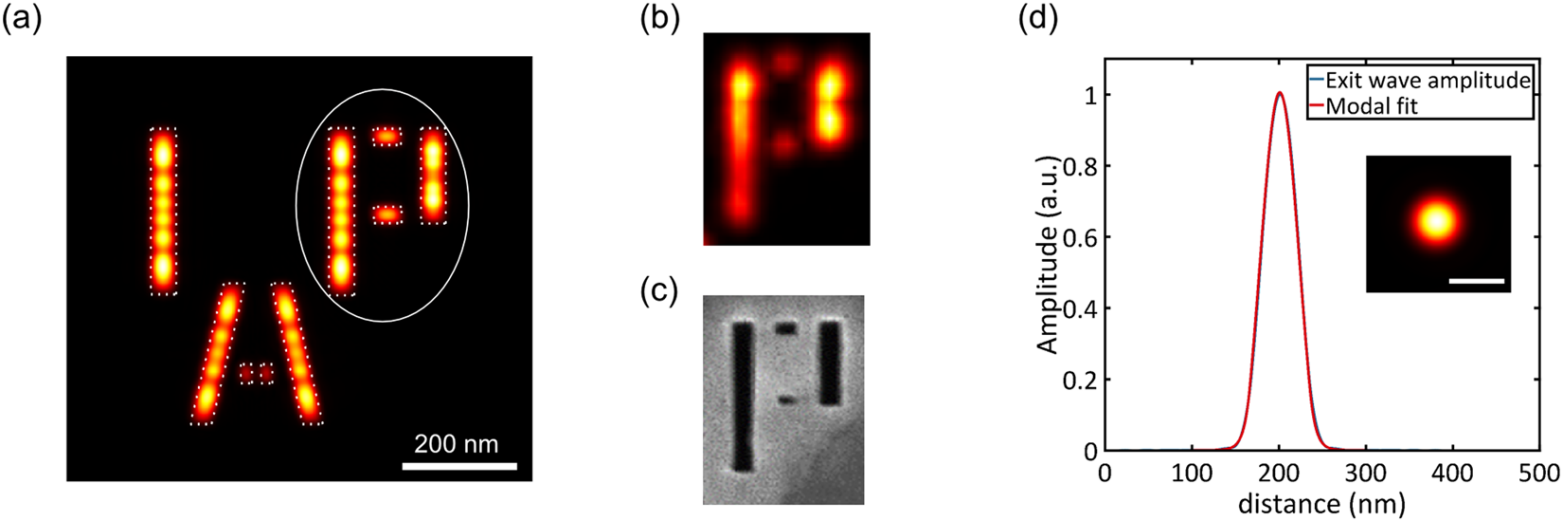
Exit surface wave (ESW) computed with the FDTD technique. (**a**) Simulated ESW of the sample showing the modulation in amplitude and varying transmissions among different features. The dotted profile shows the geometrical shape used in the simulation. (**b**) Reconstructed ESW of letter ‘P’ showing similar modulation in amplitude. (**c**) HIM picture of the back side of the sample shown for comparison. (**d**) Cross-section of the ESW amplitude and its modal fit for a simulated 50 nm diameter hole. Inset shows the ESW amplitude (scale bar is 50 nm). The cross-section is taken along the horizontal line at the center of the ESW.

A detailed look at the five reconstructed holes in Fig. 3(c) shows that their diameter (and transmission) is different among one another. The FWHM diameters vary from 48 nm to 56 nm, which matches quite well with the nominal value of 50 nm used in fabrication. The standard deviation among 12 independent reconstructions of each reference hole is ±1 nm indicating that the size of the holes can be estimated with precisions of few nm. The replica of the sample displayed in Fig. 2(c) corresponds to the reference hole with the smallest diameter of 48 nm justifying why it gave the best resolution among the five. It can also be observed in Fig. 3(d) that the image profile shows modulation along the longer bars with peaks at the edges and the letter “A” has lower transmitted amplitude. To understand the origin of these effects, a finite difference time domain (FDTD) simulation of the layered structure was performed taking into consideration the thicknesses and refractive indices of gold and Si_3_N_4_ (see Methods). The result, shown in Fig. 4, demonstrates that the exit surface wave (ESW) does not exactly follow the geometrical profile of the sample. The aspect ratio of the reference holes and the features of the sample is 1:4 making them act as a waveguide in the XUV^30^. Wavelength-sized features with high aspect ratio effectively act as a waveguide for the illuminating XUV light. The wave exiting a feature will therefore be a superposition of waveguide modes whose propagation loss depends on the feature size. In Fig. 4(a) it can be seen that the number of modulation peaks depends on the width of the feature and the ESW always has peaks at an edge. The reconstructed amplitude of the letter ‘P’ (Fig. 4b) corresponds nicely to the simulation result especially at the shorter vertical bar. Along the long vertical bar, the reduction in amplitude follows the width of the actually fabricated structure as shown from the HIM picture of the back side (Fig. 4c). In addition, every edge of the features in the simulated ESW of Fig. 4(a) appears blurred. A cross-section along the letter ‘I’ shows that the 10% to 90% resolution was limited to 21 nm although a pixel size of 0.7 nm was used in the simulation. This supports the conclusion in ref.^30^ that waveguiding putsa strict limit on the achievable resolution for features with high aspect ratio.

Note that the phase profile of the sample ESW from the simulation also shows distinct, feature-size-dependent values, which are found in the XUV image as well. These phase images contain additional information on the sample and will be subject of future investigations. A FDTD simulation of the propagation through the reference holes of 50 nm diameter (Fig. 4d) results in a non-flat-top ESW profile with a transmission efficiency of 75% (see Supplementary material). A fitting of the 2D exit wave profile using a superposition of the three dominant waveguide modes (see Supplementary material) of the reference hole structure is also shown in the cross-section of Fig. 4(d). The transmission efficiency through any feature strongly depends on its size and it goes down to only 1% for 20 nm reference holes. This explains why the reconstructed holes appear dimmer than the larger-sized features of the sample.

## Discussion

In this article, we presented a FTH experiment achieving a half-pitch resolution of 34 nm, which is the highest resolution reported so far for FTH using any light source. The robust, direct and image formation of Fourier transform holography is particularly attractive for real-world applications. Ambiguities and convergence problems, which can appear in phase retrieval-based methods such as CDI, are simply avoided although these methods can reliably be used to further increase the resolution of the holographic image. Moreover, we reduced the acquisition time for table-top systems from hours to tens of seconds by using a high-photon flux XUV source^36^, which qualifies this setup for real-time- or multidimensional studies, such as 3D imaging, spectro-microscopy or time-resolved experiments. In addition, the ability of XUV photons to penetrate through a membrane was demonstrated by resolving embedded features with a half-distance of 23 nm between one another. The achieved resolution is the highest of any table-top coherent imaging setup according to the classical Rayleigh criterion. Finally, a detailed analysis of the results and a comparison with a numerical simulation of the wave exiting the sample uncovered waveguiding effect in the XUV to be important for image formation and interpretation when approaching the wavelength-resolution limit.

Our work provides a direct route to applying FTH to real-world applications. The compact light source and imaging setup will make nanoscale imaging with XUV light available to a large scientific community. The robustness and outstanding performance of the presented holography approach will enable high resolution imaging of a large variety of real-world samples. By using a transparent Si_3_N_4_ membrane coated with a suitable absorber layer and equipped with adequate reference holes plus a transparent ~µm sized non-coated central hole, the FTH platform could be used for imaging of arbitrary samples which simply need to be placed in this central region. The robust amplitude- and phase information gained from FTH will particularly benefit imaging of weakly scattering biological objects^37,38^ or complex multi-material sample such as integrated circuits and even provide 3D images^39,40^.

Moreover, the ultrashort XUV pulse durations of HHG sources will enable real-time observation of ultrafast processes, such as electronic and magnetic dynamics, with few-femtosecond or even attosecond^41^ temporal resolution far beyond the capabilities of today’s Synchrotron facilities. In the future, even higher resolutions appear feasible by FTH utilizing even smaller reference holes and shorter XUV wavelengths. High-power fiber laser driven HHG sources with photon energies reaching the water window have already been demonstrated^14^, which opens up the possibility of imaging µm-thick biological samples, such as whole cells. Advances in laser technology will provide kW-level average powers^42^ and multi-mJ pulse energies^43^ to drive powerful XUV and soft X-ray sources and thus reduce image integration times to a minimum. At the same time more compact laser sources will be available, which potentially could even be integrated on a machine-level e.g. for online wafer inspection or a clinical environment.

## Methods

### Sample fabrication

Gold with a thickness of 200 nm was deposited on commercial 50 nm thick Si_3_N_4_ membranes (Plano, substrates with 9 membranes, each with a size of 100 × 100 µm^2^) by means of electron beam evaporation. Nanoscale patterning was done by structured milling with a helium ion microscope (HIM) Zeiss Orion plus equipped with a Fibics pattern generator. The irradiation was performed with 30 keV He^+^-ions having its maxima of projected range and nuclear energy deposition in depths close to 80 nm and 50 nm, respectively. The ion fluence was in the order of 10^19^ ions/cm^2^ for each structure using a current of the focused beam of 2 pA. Raster scanning was done in double serpentine style. Subsequently, the samples were analyzed via scanning electron microscopy (Zeiss NEON60) of both, the front and the back side.

### Experimental setup

A high power fiber laser system at 1 µm central wavelength emitting pulses with 35 fs duration, 650 µJ energy and 35 kHz repetition rate is used to drive HHG in an argon gas jet. High-order harmonics spanning the spectral range from 55–70 eV were generated with a record-high photon flux of >10^11^ photons/second for a repetition rate of 100 kHz^36^. A pair of grazing incidence plates together with aluminum filters are used to separate the driving laser from the generated XUV harmonics^16^. Two multilayer mirrors (focal lengths of 1.2 m and 300 mm) are employed to select a single harmonic as well as to focus the XUV beam on the sample. The sample is placed at the focus of the XUV beam and is illuminated from the Si_3_N_4_ side. The full-width at half-maximum (FWHM) diameter of the XUV focus is measured to be ~6 µm. In the far field, the hologram created by the interference between waves from the sample and reference hole is recorded by a CCD camera (Andor iKON L, 2048 × 2048 pixels with a pixel size of 13.5 µm). The distance between the sample and the camera is 20 mm allowing for a measurement with NA = 0.57.

### Data processing

When illuminating the opaque (unstructured) part of the sample, close to half as many peak counts compared to the hologram were recorded for the few central pixels showing that this opaque part still transmits some of the XUV light. A Gaussian function with the estimated width and peak count of this directly transmitted beam was subtracted from the few central pixels of the hologram to compensate for this issue. Hot pixel removal and curvature correction^44^ were then performed on the hologram.

### Phase retrieval

The oversampling ratio of the measurement is 7 and the size of the object to be reconstructed is 2.5 µm as this includes the reference holes. The sampling period of the recorded hologram (which equals the product of the binning and pixel size) is inversely related to the field of view of the imaged object. In our case, the sampling period (effective pixel size) of the recorded hologram is 54 µm (actual pixel size of 13.5 µm and binning of 4) which results in a total field of view of 8 µm. But this field of view consists of both the autocorrelation and the multiple cross-correlation terms in the image. The sampling period is chosen to achieve an oversampling ratio greater than 2, for the sample area including all reference holes, which is necessary for successful phase retrieval. Summing up multiple diffraction patterns (frames) increased the dynamic range of the recorded hologram which enabled a further resolution improvement by iterative algorithms. The guided RAAR algorithm^34,35^ with a β-parameter of 0.95, 12 independent reconstructions and 5 generations were used in performing the phase retrieval. The shrink-wrap algorithm^45^ was used to update the support of the sample and low thresholds were used not to over-shrink the features. Since the reference wave has lower intensity than the exit wave from the object, a lower threshold is used for the region outside the seeded object and this region includes the reference holes.

### FDTD simulations

The numerical simulations were performed using a commercial FDTD solver (Lumerical FDTD solutions 2016b). The sample was simulated as a three-dimensional structure with the 200 nm thick gold layer on a 50 nm thick Si_3_N_4_ membrane. The geometry of the IAP logo was created from the front side HIM picture of the milled structure. The reference hole was simulated as a perfectly circular structure. The simulated membrane area was 1.5 µm × 1.5 µm in case of the IAP logo and 0.7 µm × 0.7 µm in case of the reference hole. The etched structures were centered within this area.

A uniform grid with a resolution of 0.714 nm was used to discretize the geometry. Staircasing effects were reduced by using Lumerical’s conformal mesh refinement technology (variant 1). The structure was excited from the Si_3_N_4_ side by a normally incident pulsed plane wave total-field/scattered-field source with a center wavelength of 18.1 nm and a bandwidth of 2 nm. A plane wave illumination was assumed which seems reasonable in view of the large spot size of the experimental beam. The simulated polarization was the same as in the experiment: linear polarization tilted by −45° with respect to the x-axis. Stretched coordinate perfectly matched layers (PMLs) were employed on all boundaries to absorb the outgoing scattered fields. The transmitted total electric and magnetic fields at a wavelength of 18.1 nm were sampled 1 nm behind the gold layer with a frequency domain field monitor.

The material data of the Au and Si_3_N_4_ were obtained from the X-ray database of the Center of X-Ray Optics^46,47^. The relative permittivity from the tabulated data was approximated by the sum of 5 (Au) and 4 (Si3N4) Lorentz resonances, respectively, within the spectral range of the source.

### Data availability

The data that support the plots within this paper and other findings of this study are available from the corresponding author upon reasonable request.

## Electronic supplementary material

Supplementary Information
