# Supplementary material for: High resolution XUV Fourier transform holography on a table top
Source: Sci Rep. 2018 Jun 6;8:8677. doi: 10.1038/s41598-018-27030-y (PMC5989263; doi:10.1038/s41598-018-27030-y)
Supplement: Supplementary file 1 — Supplementary Information [file 41598_2018_27030_MOESM1_ESM.docx]

High resolution XUV Fourier transform holography on a table top

G.K. Tadesse^1,2^, W. Eschen^2^, R. Klas^1,2^, V. Hilbert^2^, D. Schelle^2^, A. Nathanael^2^, M. Zilk^2^, M. Steinert^2^, F. Schrempel^2^, T. Pertsch^2^, A. Tünnermann^1,2,3^, J. Limpert^1,2,3^ and J. Rothhardt^1,2,*^

^1^Helmholtz-Institute Jena, Fröbelstieg 3, 07743 Jena, Germany

^2^ Institute of Applied Physics, Abbe Center of Photonics, Friedrich Schiller University Jena, Albert-Einstein-Straße 15, 07745 Jena, Germany

^3^Fraunhofer Institute for Applied Optics and Precision Engineering, Albert-Einstein-Str. 7, 07745 Jena, Germany

*Corresponding author: [jan.rothhardt@uni-jena.de](mailto:jan.rothhardt@uni-jena.de)

**Supplementary Material**

# **FDTD Simulations**

The numerical simulations were performed using a commercial FDTD solver (Lumerical FDTD solutions 2016b [1]). The sample was simulated as a three-dimensional structure with the 200nm thick gold layer on a 50 nm thick Si_3_N_4_ membrane. The reference hole was simulated as a perfectly circular structure and the simulated membrane area was 0.7 µm x 0.7 µm. The etched structures were centered within this area.

A uniform grid with a resolution of 0.714 nm was used to discretize the geometry. The structure was excited from the Si_3_N_4_ side by a normally incident pulsed plane wave total-field / scattered-field source with a center wavelength of 18 nm and a bandwidth of 2 nm. The polarization of the source was -45° with respect to the x-axis. The transmitted total electric and magnetic fields at a wavelength of 18.1 nm were sampled 1 nm behind the gold layer with a frequency domain field monitor (See Methods for details).

The transmission efficiency of the hole was calculated as the the power transmitted through the hole divided by the incident intensity multiplied with the geometrical hole area:

$$q_{T}=\frac{4P_{T}}{{\pi d^{2}I}_{s}}$$

where $d$ is the hole diameter. The dependence of the transmission efficiency on the hole diameter is illustrated in Figure 1.


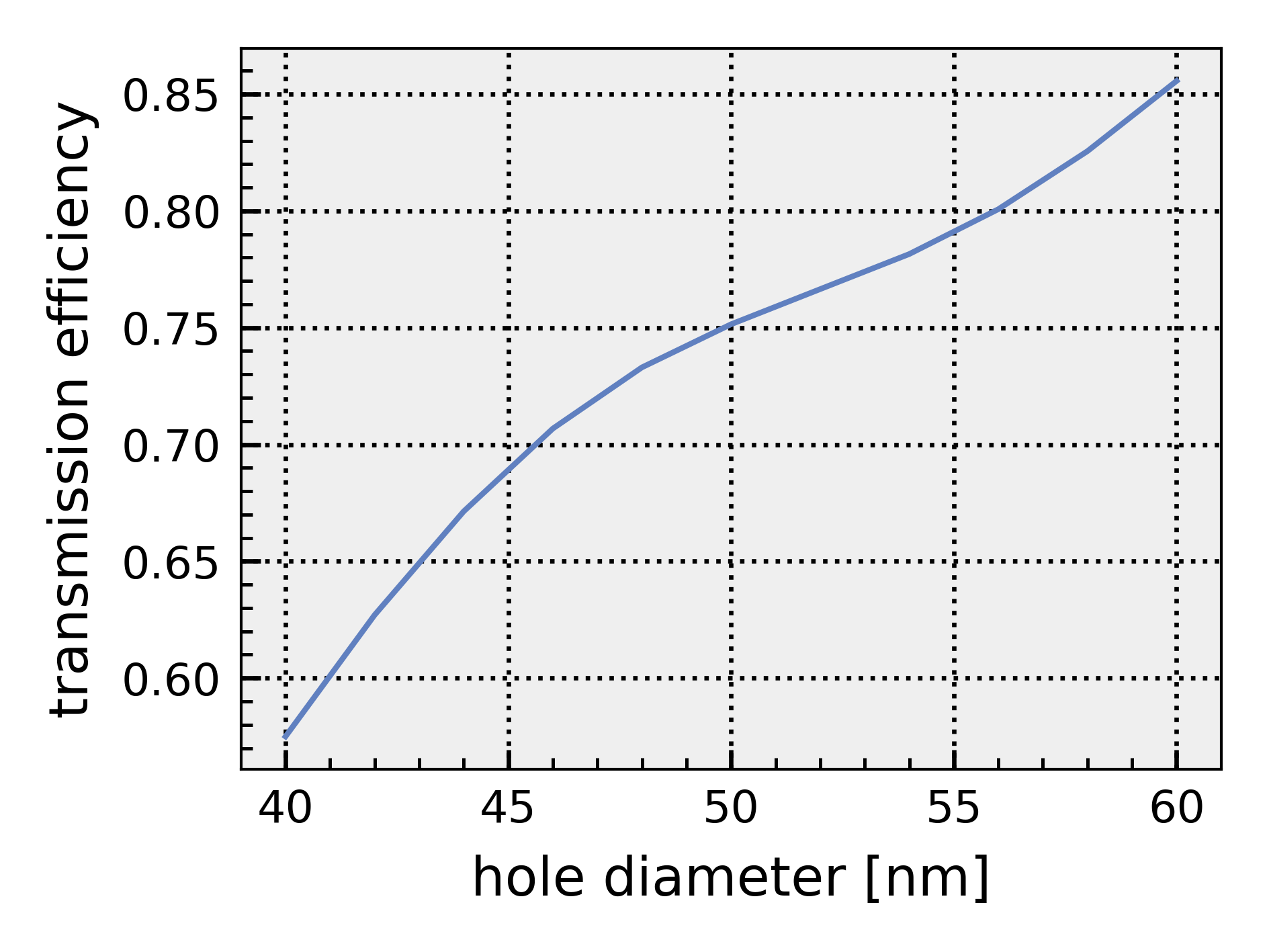


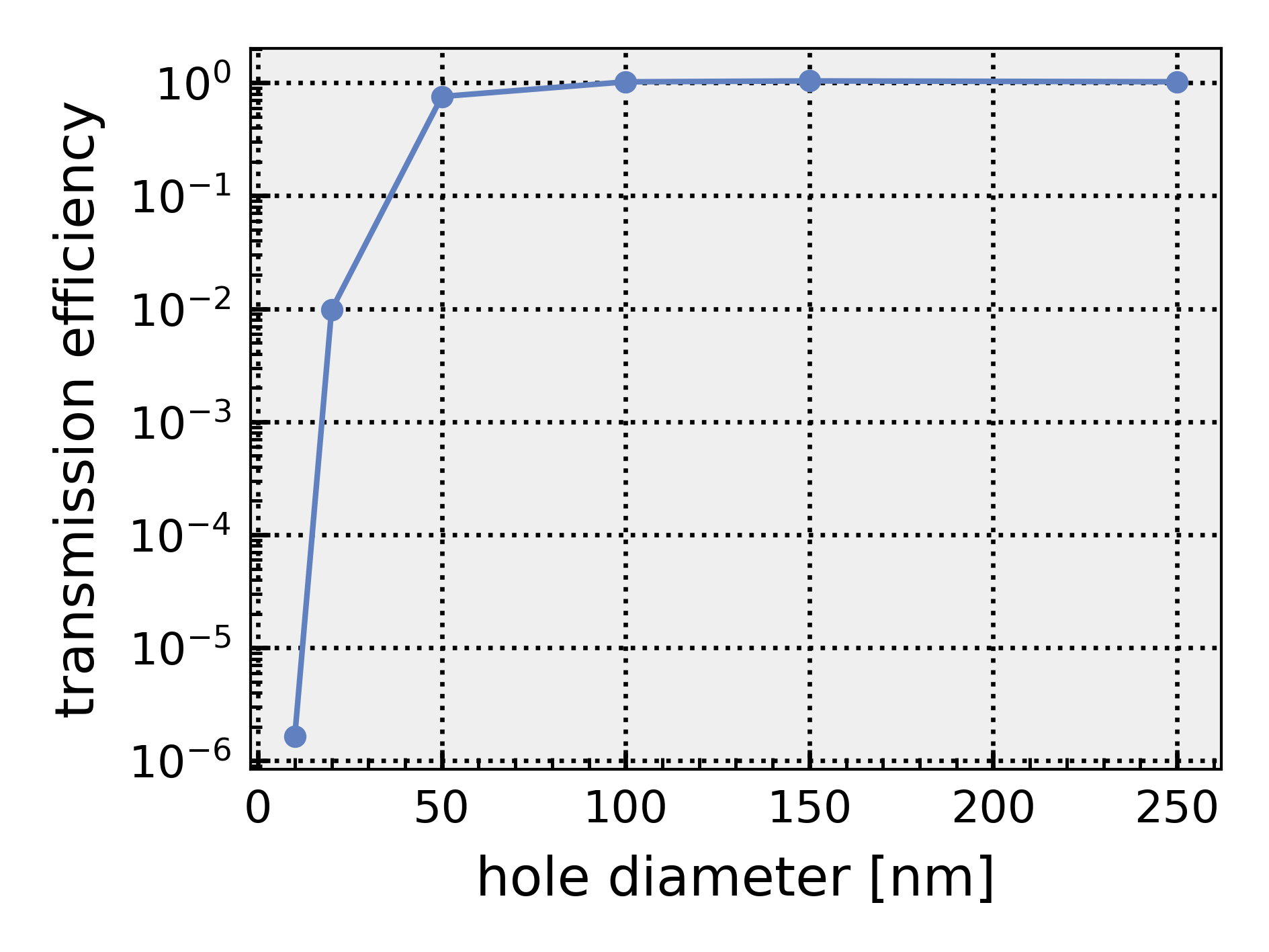


Figure 1: Transmission efficiency in dependence of the hole diameter.

# **Mode analysis**

The waveguide modes of a hole in a gold layer were calculated with a commercial finite-difference mode solver (Lumerical Mode Solutions 2017b [2]). The discretization was the same as for the FDTD simulations. The gold refractive index was taken from the fitted model that was used in the FDTD calculations.

At a wavelength of $\lambda=18.1 \mathrm{nm}$ a hole with a diameter of $d=50 \mathrm{nm}$ supports 12 waveguide modes, 2 non-degenerate modes and 5 modes with a twofold degeneracy. At normal incidence only three modes can be excited with linearly polarized light for symmetry-reasons. These modes are shown in Figure 2.

The exit wave from the FDTD simulation of the hole was expanded into the waveguide modes using the built-in facilities of Lumerical Mode Solutions (namely the expand2 function [3]). The following expansion coefficients were obtained (the hole was excited with a polarization of 45° with respect to the x-axis, hence both of the degenerate modes are excited, as the polarization of the modes was axis aligned):

| mode | $n_{\mathrm{eff}}$ | expansion coefficient |
| --- | --- | --- |
| 1 | $0.971212 + 0.004048j$ | $-(0.880946 + 0.475330j)$ |
| 2 | $0.971212 + 0.004048j$ | $-(0.880946 + 0.475330j)$ |
| 7 | $0.870005 + 0.022613j$ | $0.008306 + 0.029270j$ |
| 8 | $0.870005 + 0.022613j$ | $0.008302 + 0.029267j$ |
| 11 | $0.850050 + 0.035951j$ | $-(0.045352 + 0.063200j)$ |
| 12 | $0.850050 + 0.035951j$ | $-(0.045350 + 0.063198j)$ |

Table 1. Expansion coefficient for the different waveguide modes

The FDTD fields are shown in Fig. 2 and the residuals after the mode expansion are shown in Figure 3.


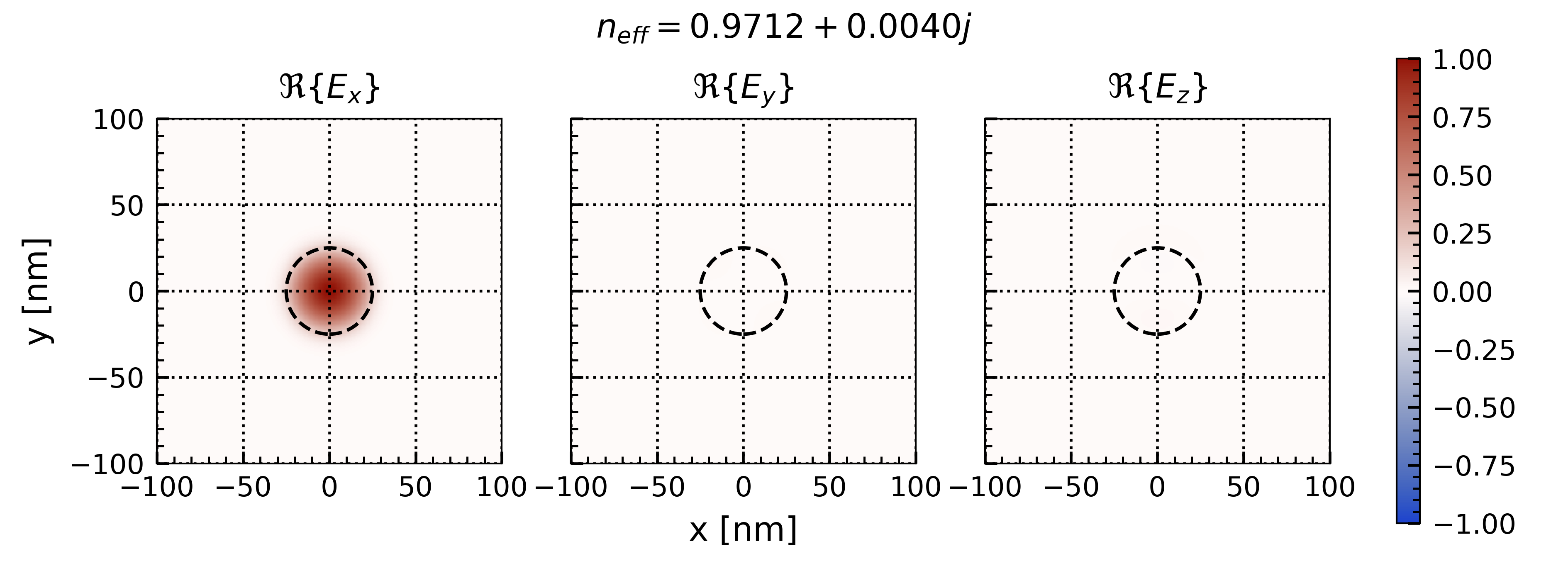

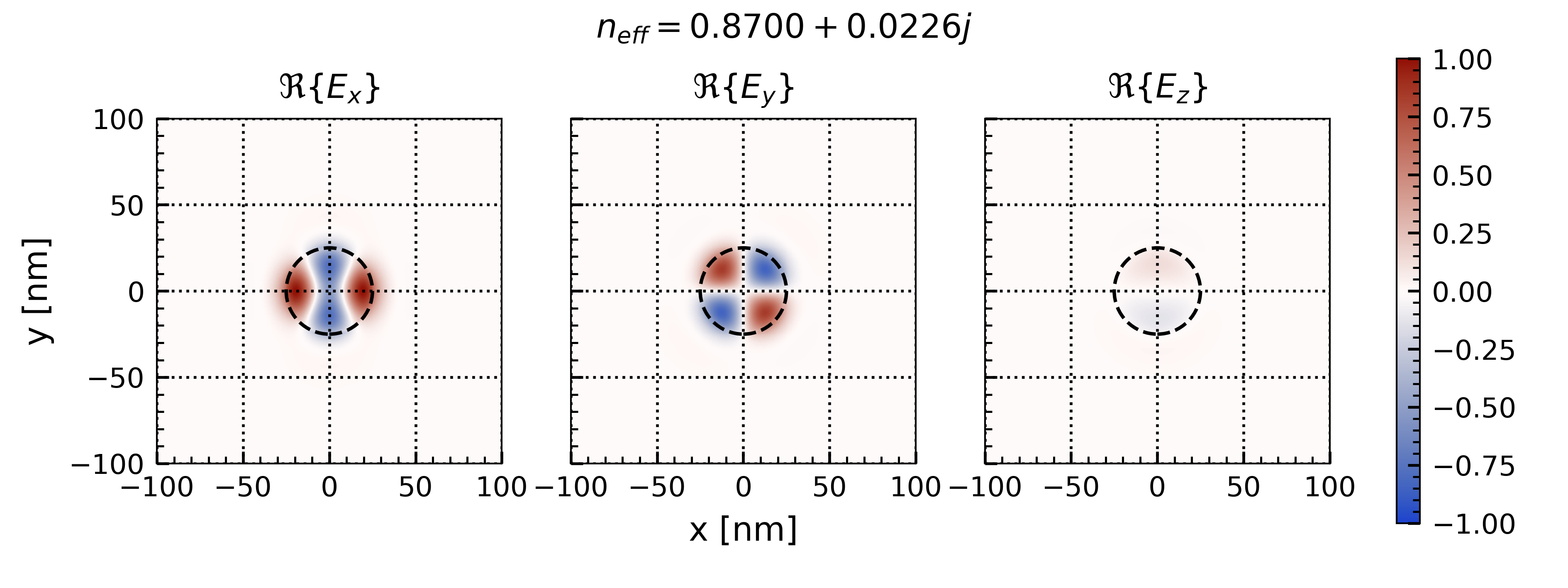

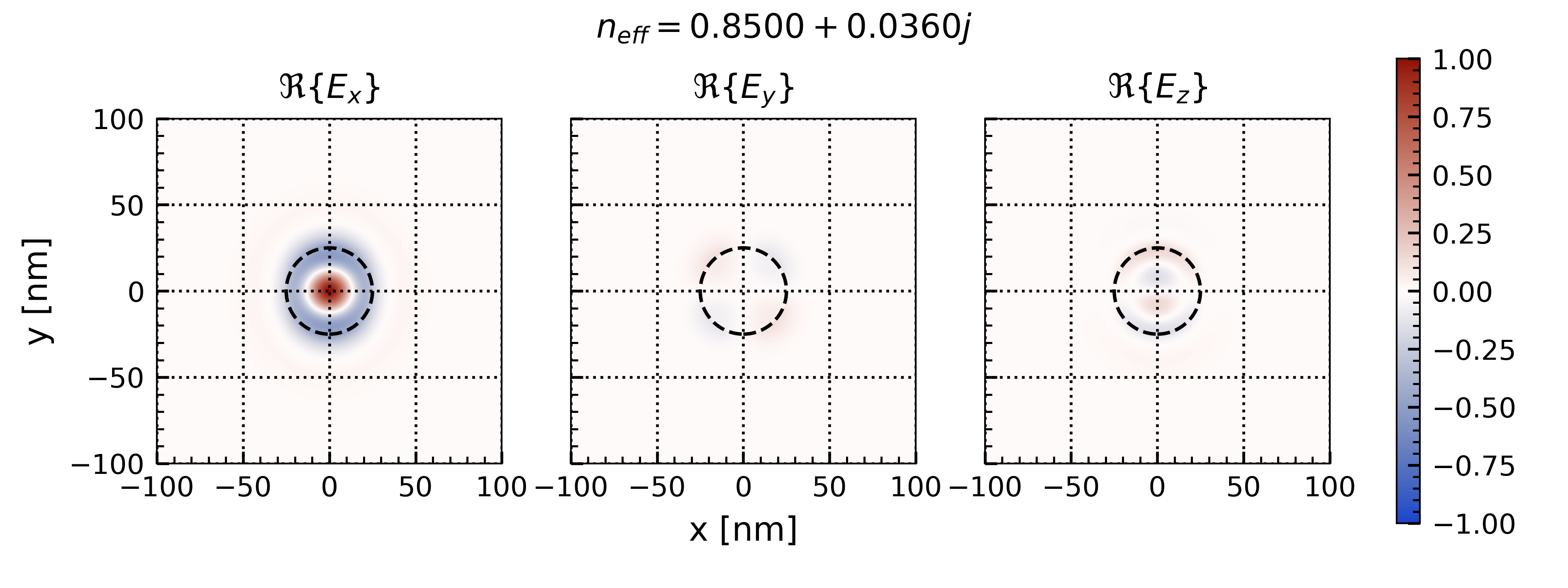


Figure 2: Waveguide modes that can be excited with linearly x-polarized light at normal incidence.


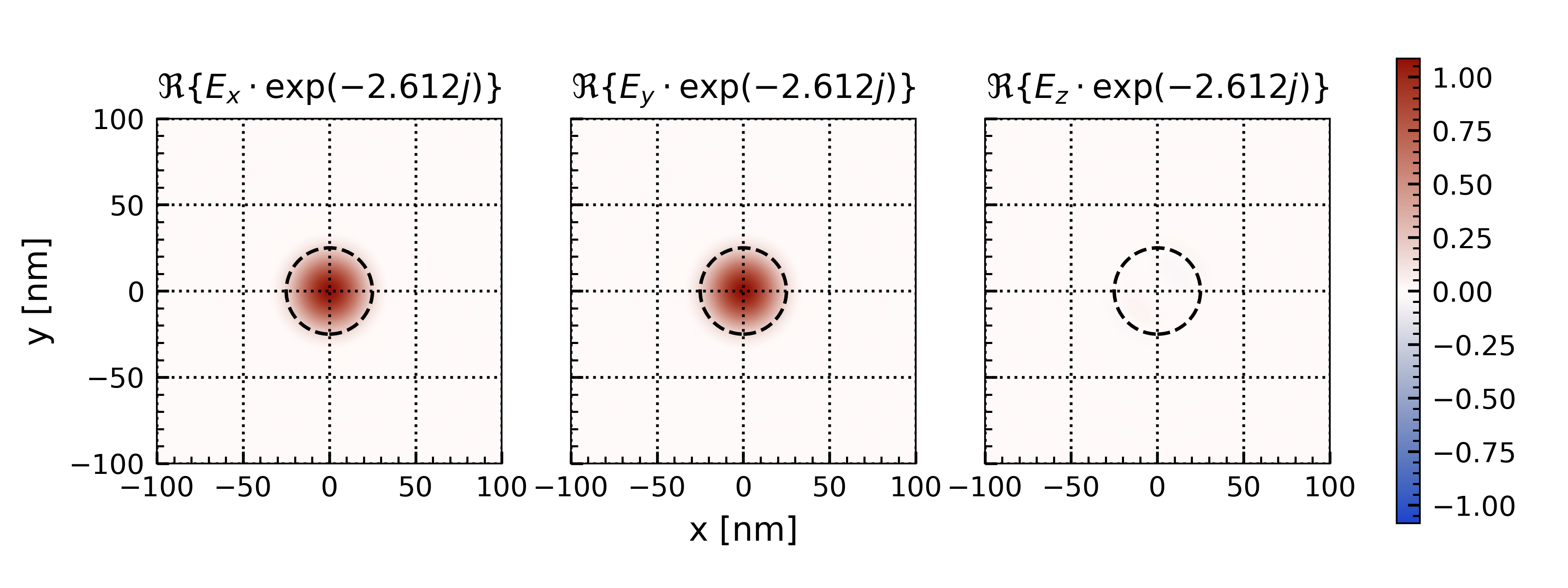

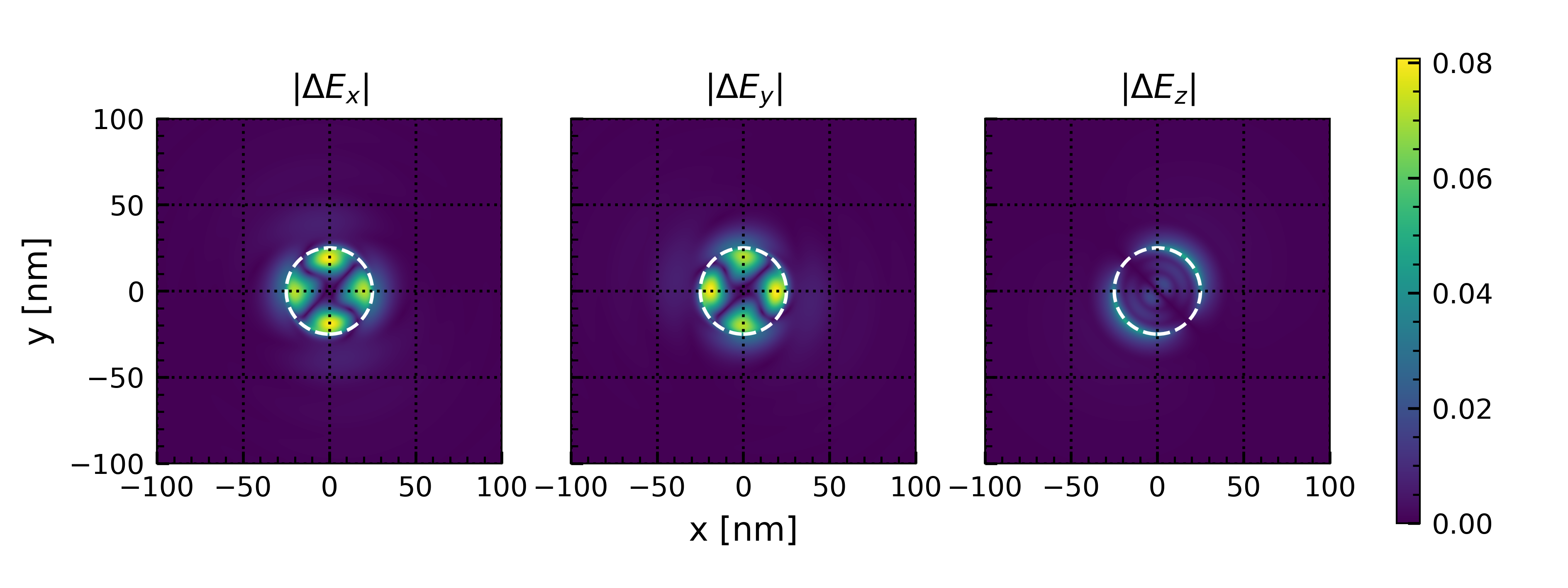


Figure 3: Exit wave of a $d=50 nm$ hole from a FDTD simulation and residuals after decomposition into waveguide modes.

# References

[1] https://www.lumerical.com/tcad-products/fdtd/

[2] https://www.lumerical.com/tcad-products/mode/

[3] https://kb.lumerical.com/en/ref_scripts_expand2.html
